# Clinical translation and landscape of copper nanoparticles

**DOI:** 10.1007/s13346-026-02094-w

**Published:** 2026-03-07

**Authors:** Regina D. Hadiwinata, Rui Zhang, Roman A. Barmin, Fabian Kiessling, Twan Lammers, Roger M. Pallares

**Affiliations:** Institute for Experimental Molecular Imaging, RWTH Aachen University Hospital, 52074 Aachen, Germany

**Keywords:** Copper nanoparticles, Clinical translation, Nanomedicine, Wound healing, Antimicrobial

## Abstract

Although the medical use of copper dates back millennia and medicines based on copper nanoparticles have been employed for over a century, their benefits are still often questioned. Over the past three decades, advances in nanotechnology have renewed interest in copper-based nanoformulations for healthcare. While most preclinical studies have focused on systemically administered copper nanomedicines for a broad range of therapeutic purposes, clinical trials remain scarce and are primarily centered on topical applications, where long-term off-target accumulation can be minimized to mitigate safety concerns. Despite exhibiting only moderate antiseptic activity compared with other inorganic nanomaterials, copper nanoparticles demonstrate significant wound healing potential through complex mechanisms involving angiogenesis promotion and stabilization of extracellular skin proteins. In this work, we discuss the current landscape of copper nanoparticles (and related microparticulate structures), and we thoroughly analyze the characteristics that have enabled their translation and use in clinical settings.

## Introduction

Copper is a metal that has been recognized and exploited for its health benefits since antiquity [1]. Ancient Egyptian texts (2600–1500 BC) described the use of copper to sterilize wounds and recommended copper compounds for treating skin conditions, such as infections, burns, and irritations. Similarly, the Greek *Hippocratic Corpus* (fifth century BC) suggested applying copper-based treatments to leg ulcers and other conditions [2]. During the nineteenth century, the medical use of copper gradually expanded, supported by emerging scientific evidence, such as the lower incidence of cholera among copper miners [3]. This renewed interest coincided with the rise of colloidal silver as a potent antiseptic towards the end of the century, with several nanoformulations, such as Collargol and Protargol [4, 5], achieving (relative) widespread use in eye infection and wound antisepsis treatments before the antibiotic era [6]. Likewise, in the early twentieth century, colloidal copper formulations such as Cuprargol/Cuprargolum (copper-albumin colloids [7, 8]) were explored for antimicrobial applications, though these proved less successful than their silver counterparts. Other copper nanoformulations, including Cuprase [9, 10] and Electrocuprol [11], were even explored for systemic treatments against cancer and tuberculosis but were eventually abandoned due to poor clinical efficacy. Over the past three decades, advances in nanoscience and nanotechnology have rekindled interest in copper colloids, leading to the development of new commercial formulations, particularly for wound healing and antimicrobial topical applications and coatings.

Copper (and copper oxide) nanoparticles (CuNPs) exhibit physicochemical properties different from those observed in ionic or bulk metallic copper [12]. For instance, their large surface-to-volume ratios enhance reactivity and support unique interactions with biological systems, contributing to antimicrobial activity [13]. Several mechanisms are thought to contribute to these effects, including: (i) the gradual release of Cu^2+^ ions, which can cause membrane damage, protein denaturation, and metabolic disruption; (ii) the generation of reactive oxygen species, inducing oxidative stress, lipid peroxidation, DNA strand breaks, and protein oxidation; and (iii) direct physical interactions with membranes, leading to structural deformation and leakage of cellular content [14–16]. The extent of these antimicrobial effects depends strongly on nanoparticle features, such as oxidation state, size, morphology, composition, and surface chemistry, features that can be precisely tuned during their synthesis and formulation [17, 18]. As a result of these properties, CuNPs have been incorporated into various consumer and daily-use products, including antimicrobial coatings, textiles, and cosmetics. However, their use remains niche, and commercially available information tends to be limited.

One aspect in which CuNPs are stronger than other inorganic colloids is in their ability to promote wound healing through multiple synergistic mechanisms [19]. In addition to their antiseptic activity, copper ions released from the colloids facilitate skin regeneration, angiogenesis, and stabilization of extracellular skin proteins. For example, copper promotes the induction of vascular endothelial growth factor and stimulates angiogenesis by enhancing the expression of hypoxia-inducible factor-1-alpha [20]. Furthermore, copper ions are essential cofactors for the function of key enzymes, such as lysyl oxidase, superoxide dismutase, and cytochrome c oxidase, which play crucial roles in maintaining vascular integrity and supporting angiogenesis [21–23].

Interestingly, CuNPs made of metallic copper also exhibit localized surface plasmon resonances, collective oscillations of conduction-band electrons induced by excitation with electromagnetic waves of specific wavelengths [24]. These resonances give rise to strong extinction coefficients in the visible and near-infrared range [25, 26], similar to those displayed by gold and silver nanoparticles [27–32]. However, copper is prone to oxidation, forming copper oxide that does not support plasmonic resonances. This tendency towards surface oxidation, combined with its weaker and broader plasmonic bands [26], has limited the use of CuNPs as optical agents in biosensing applications [33]. Nevertheless, their relatively strong photothermal responses have been explored for (pre)clinical photothermal applications [34, 35], although such applications remain less developed than those based on gold and silver nanostructures [6, 36–40].

Nowadays, only a few therapeutic CuNP formulations have been explored in clinical settings, despite a rapidly growing body of preclinical research. Several factors contribute to this gap between preclinical research volume and clinical translation. First, while many new copper nanoconstructs demonstrate scientific innovation, they often fail to address unmet clinical needs or to outperform established therapeutic standards [41]. Second, preclinical studies frequently emphasize multifunctionality, resulting in overly complex nanostructures composed of multiple components whose biological behavior cannot be reliably predicted, unlike clinically approved nanomedicines, which are typically far simpler in composition and design [42–45]. Third, the synthesis and scale-up of such formulations is problematic, as many can only be produced in small quantities under tightly controlled conditions [46]. Fourth, as with other inorganic nanomaterials, there are ongoing concerns regarding long-term retention, biodistribution, and potential toxicity [47–51]. Some of these risks can be minimized by surface functionalization with biocompatible polymers, such as polyethylene glycol, and by restricting their use to localized applications, thereby avoiding systemic administration.

**Fig. 1:**
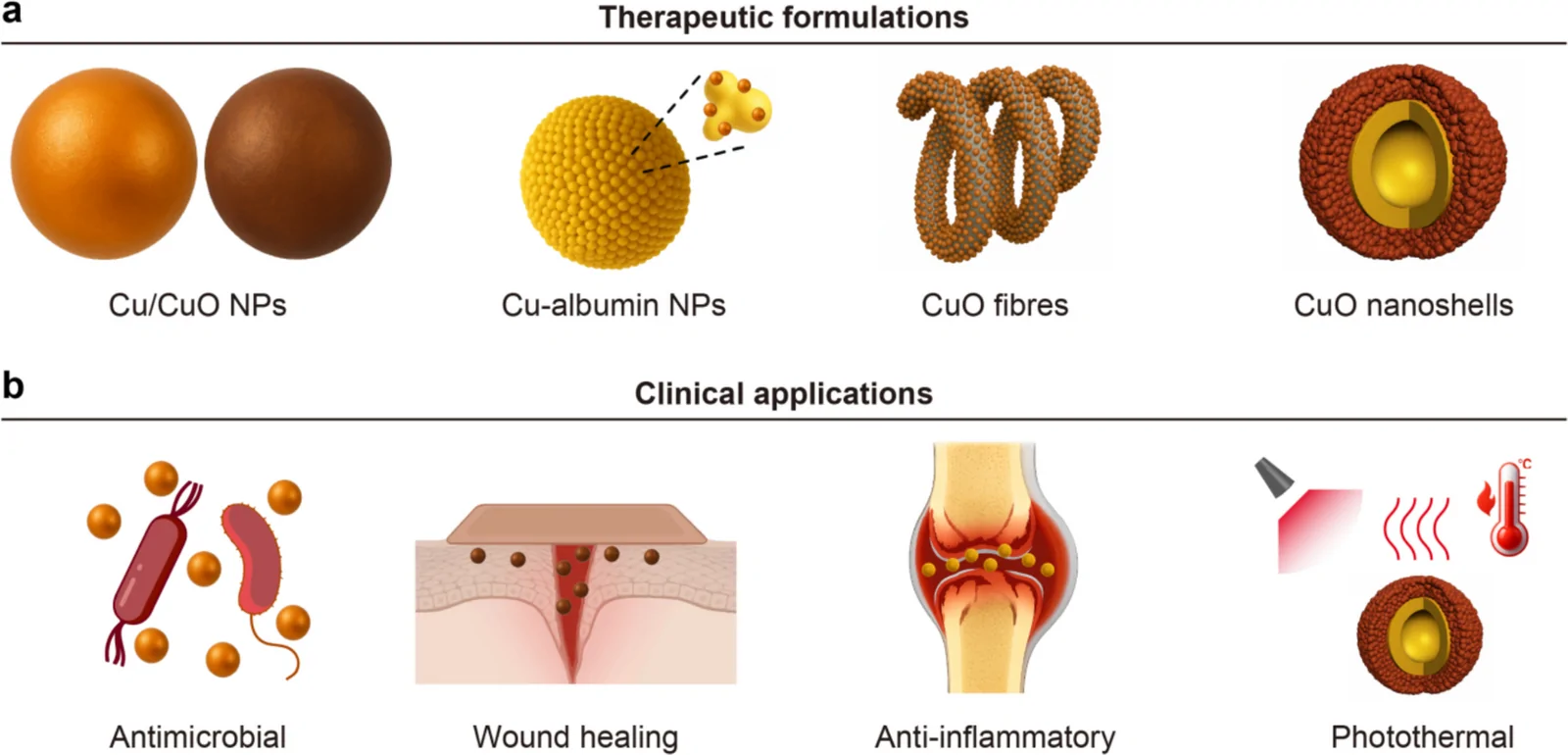
Fundamentals of clinical copper nanoformulations. (**a**) Schematic illustration of therapeutic copper (nano)formulation and (**b**) their clinical applications

In this analysis, we examine the current clinical landscape of therapeutic CuNPs (Fig. 1) and, when relevant, related microparticulate formulations (Table 1). By analyzing copper constructs under clinical investigation, we identify the key physicochemical and translational features that have enabled their translation and highlight the therapeutic contexts in which CuNPs can offer medical benefit.

**Table 1:** Therapeutic copper nanoformulations in clinical trials

Colloid type	Oxidation state	Administration	Application	Clinical trial
Bare CuNPs	0	Topical	Allergic syndrome	NCT06529913, Active, not recruiting yet (2024 –)
CuNPs (150–350 nm)	0	-	Antimicrobial	NCT04775238, Status unknown (2021 –)
CuO colloid dressings	+ 2	Topical	Wound healing therapy in obstetric wounds	NCT03284749, Completed (2016–2017)
CuO colloid dressings (MedCu)	+ 2	Topical	Wound healing therapy in pressure ulcers, post-operation wounds, and diabetic foot wounds	NCT04963998, Completed (2019–2020) NCT04634838, Terminated (2021–2023) NCT05215730, Completed (2021–2023)
CuO fiber fabrics	+ 2	Topical	Prevention of chemotherapy-induced hand-foot syndrome	NCT01291628, Status unknown (2012 –)
CuO colloids garments	+ 2	Topical	Treatment of lipodermatosclerosis	NCT03283800, Completed (2015–2017)
Copper-albumin nanoparticles	+ 2	Topical	Treatment of knee osteoarthritis	NCT03736109, Status unknown (2017 –)
Cupriferous hollow nanoshells	+ 1	Topical	Photothermal therapy against microbial keratitis	NCT05268718, Phase I, Status unknown (2021 –)

## Clinical landscape of copper nanoparticles

### General toxicity and safety profile

Metal colloids have become increasingly common as over-the-counter products, marketed both as oral nutraceutical supplements and topical creams [52]. Numerous copper colloid formulations are commercially available, often advertised with purported benefits such as enhanced enzymatic and metabolic activity, improved immune function, and overall enhanced well-being. However, most of these claims are not supported by scientific evidence. At the same time, concerns persist regarding the biological fate, long-term accumulation, and potential chronic toxicity of these materials [53], particularly in the case of unfunctionalized colloids used as nutraceuticals [54].

CuNP toxicity is highly dependent on both dose and exposure duration. Acute high local concentrations can trigger oxidative stress, membrane damage, and cytotoxicity [55, 56]. In contrast, chronic low-dose exposures have been associated with progressive tissue accumulation, particularly in highly perfused and fenestrated organs, such as the liver and spleen, resulting in sustained redox imbalance [57]. Toxicological outcomes are also strongly influenced by the route of administration. Topical applications generally produce localized exposures with limited systemic absorption [58]. In contrast, intravenously administered colloids are rapidly sequestered by the reticuloendothelial system, leading to significant accumulation in the liver and spleen, requiring thorough evaluation of long-term effects [59].

Systemically released copper ions interact (and potentially disrupt) tightly regulated homeostatic pathways. The transporters ATP7A/B control intracellular distribution and biliary excretion [60], while ceruloplasmin serves as a major copper-binding protein in circulation [61]. Nevertheless, CuNPs can overwhelm or bypass these regulatory mechanisms, if the release of ions exceeds endogenous buffering capacity, increasing the risk of hepatic overload and injury.

Despite promising preclinical data, the safety profile of most CuNP formulations remains incompletely characterized. Key gaps include long-term biodistribution, copper speciation under physiological conditions, endogenous ion-release kinetics, potential disruption of copper homeostasis, and immunotoxicity. Addressing these clinical gaps will be essential to define safe therapeutic windows and meet regulatory requirements for future clinical translation.

In this context, in late 2024, the Policlinico Agostino Gemelli (Italy) launched an interventional clinical trial (NCT06529913) to investigate the effect of unfunctionalized colloidal nanoparticles, including metallic CuNPs, on various allergic syndromes. The study involves controlled colloidal exposure through patch testing in both healthy participants and patients diagnosed with allergic contact dermatitis or systemic allergic syndromes. Outcomes will include the assessment of skin responses, such as papule formation and erythematous reaction. In addition, respiratory function will be evaluated using tidal breathing analysis with oscillometry, while metal concentrations will be quantified in serum and urine samples. Biomarkers linked to heavy metal toxicity, including protein oxidation products and serum zonulin levels, will also be monitored. Although patient recruitment has not yet begun, the study anticipates enrolling around 280 individuals, comprising both healthy volunteers and affected patients.

### Copper nanoparticles as antimicrobial and wound healing agents

Cuprargol (also known as Cuprargolum) was the earliest known copper-based nanoformulation developed for clinical use. In contrast to current copper-based nanoformulations, which are typically based on metallic copper or copper oxide cores, Cuprargol consisted of a colloidal protein-metal complex, in which ionic copper was stabilized through binding to albumin [7, 8]. Inspired by the prior success of colloidal silver antiseptics, Cuprargol gained popularity between 1895 and 1915 for treating ophthalmic infections (e.g. conjunctivitis) and urogenital diseases (e.g. gonorrhea). The albumin carrier markedly reduced the causticity of copper salts while preserving their antimicrobial efficacy. Nevertheless, with the development of modern antibiotics, such as sulfonamides, Cuprargol use rapidly declined, and the formulation eventually disappeared from the market, unlike its silver analogues (e.g. Collargol and Protargol), which remain commercially available as over-the-counter topical antiseptics.

Silver nanoparticles are generally regarded as more potent broad-spectrum antiseptics; however, CuNPs are considerably more cost-effective [62, 63]. Currently, an ongoing clinical trial (NCT04775238) led by Sohag University (Egypt) is directly comparing the antimicrobial performance of CuNPs and silver nanoparticles against biofilms formed by antibiotic-resistant nosocomial pathogens, such as *Staphylococcus aureus* and *Pseudomonas aeruginosa*. The outcomes of this study are expected to provide valuable insights into the relative efficacy of both nanoconstructs as next-generation topical antimicrobial agents.

While the antiseptic capabilities of CuNPs are moderated compared with other inorganic nanoparticles, they exhibit markedly stronger wound healing effects through multiple synergistic pathways. Copper ions released from the nanoparticle cores modulate various growth factors and cytokine signaling mechanisms [64], including the induction of vascular endothelial growth factor and the upregulation of hypoxia-inducible factor-1-alpha, both of which support angiogenesis [20]. Moreover, copper is an essential trace element that serves as a cofactor for several enzymes, such as lysyl oxidase, superoxide dismutase, and cytochrome c oxidase, which regulate extracellular-matrix crosslinking, endothelial migration, vascular integrity, and pro-angiogenic signaling [21–23].

Currently, CuNPs are rarely applied in liquid formulations; instead, most clinical studies focus on their incorporation into wound dressings. A notable example is the *Effect of Copper on the Healing of Obstetric Wounds (ECHO)* interventional trial (NCT03284749), sponsored by the National Health Service Trust (UK). The study evaluated the efficacy of copper oxide particulates-impregnated dressings in promoting healing of obstetric wounds, particularly perineal tears and caesarean sections.

Perineal tears occur in around 80% of vaginal deliveries and often require suturing [65]. Up to 10% of these wounds develop infections that delay recovery and hinder postpartum care [66]. Given that copper colloids have been reported to have antimicrobial and pro-angiogenic properties, maternity sanitary pads containing 3% copper oxide particles (developed by Copper Clothing Ltd) were explored in a double-blind, randomized trial involving 450 participants. Notably, the incidence of wound infection decreased from 37.4% in the control group to 8.6% in the copper-treated group [67].

A second arm of the *ECHO* trial evaluated the copper oxide colloids-impregnated dressings for caesarean section wounds. A total of 324 women were enrolled in the study and randomized into two groups: copper-oxide impregnated wound dressing and non-copper wound dressing (control group). Surgical site infections occurred in 29.7% of the control group versus 18.2% of the copper group, corresponding to a 38.7% relative risk reduction (*p* < 0.05) [68]. The effect was especially notable for (deep) organ/space infections, which occurred in only 2.5% of the copper group compared with 12.7% of the control group (*p* < 0.005). No adverse effects or allergic reactions were reported. Overall, the *ECHO* trial provides clinical evidence that copper oxide-impregnated dressings significantly reduce postpartum wound infections following both vaginal and caesarean deliveries.

**Fig. 2:**
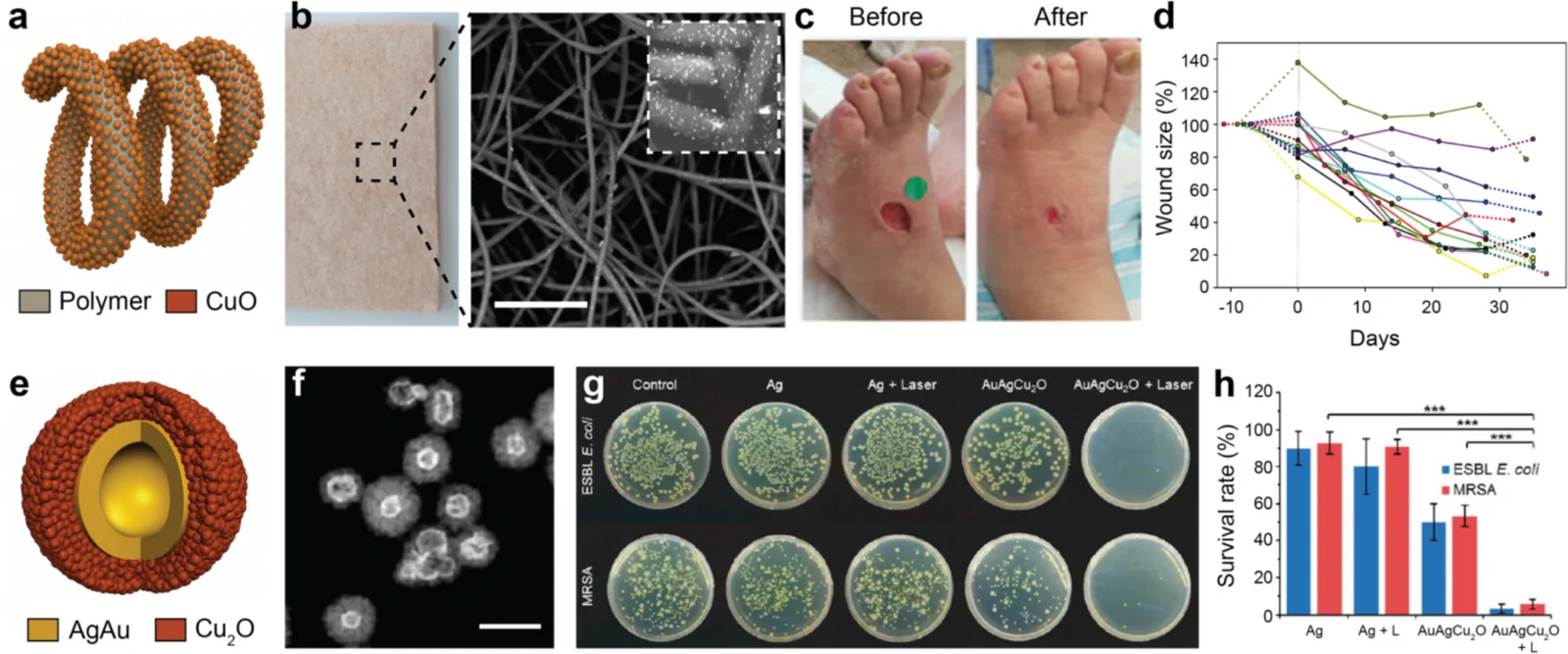
Representative therapeutic copper nanoconstructs explored clinically. (**a**) Schematic illustration of polymeric fibres coated with CuO colloids used in MedCu dressings. (**b**) Photograph of a MedCu dressing alongside scanning electron micrographs at two magnifications showing the fibre architecture and CuO colloid distribution (scale bar: 0.3 mm). (**c**) Representative images of a diabetic wound before and after MedCu treatment. (**d**) Clinical response curves of diabetic patients treated with MedCu dressings. Adapted with permission of Refs [74] and [75]. Copyright 2021 MDPI. (**e**) Schematic depiction and (**f**) dark-field transmission electron micrograph (scale bar: 100 nm) of cupriferous hollow nanoshells used to treat microbial keratitis. (**g**) Representative microorganism colonies following treatments with silver nanoparticles (Ag), near-infrared laser (L), and/or hollow nanoshells (AgAuCu_2_O). (**h**) Quantitative histograms showing bacterial survival rates under the different treatment conditions. Adapted with permission of Ref [103]. Copyright 2020 American Chemical Society

Another prominent example is MedCu, a commercially available line of wound dressings developed by MedCu Technologies (Israel) and distributed worldwide. These are single-use, multilayer dressings containing an internal absorbent layer of polyester and cellulose impregnated with copper oxide microparticles (Figs. 2a and 2b). The copper oxide enables sustained ion release into the wound environment, providing both antimicrobial activity and wound healing support for up to seven days. Furthermore, using microparticles rather than nanoparticles reduces the risk of unwanted tissue penetration, thereby limiting systemic copper exposure. The safety and efficacy of MedCu dressings have been investigated in three recent clinical trials and several case studies, with results from two trials already published.

The first published study (NCT04634838), initiated in 2021, was a pilot efficacy trial comparing MedCu dressings to metallic silver-based dressings in patients with pressure ulcers or postoperative wounds. The study design consisted of two phases: initially, patients were treated with silver dressings for up to three weeks; if the wound area did not decrease by at least 50%, treatment was switched to MedCu for an additional three weeks. After enrolling 20 patients, the trial was terminated early due to the significantly superior outcomes with MedCu, which achieved a 2.4-fold greater reduction in wound area (87% vs 37%) compared to silver dressings [69]. Based on these positive results, the researchers discontinued using the silver dressing and began applying the MedCu dressing from the start of the treatment.

In a follow-up trial (NCT05215730), completed in 2023, a randomized, multisite, two-arm study evaluated the efficacy of the MedCu dressing compared with negative pressure wound therapy (NPWT) for treating diabetic foot wounds. Diabetic foot infections, which often arise from infected diabetic foot ulcers, can lead to severe complications, including gangrene, necrotizing fasciitis, or osteomyelitis [70]. Standard treatment frequently involves extensive surgical debridement and, in severe cases, partial foot amputation. Once the infection is controlled, wound closure becomes essential. NPWT is commonly used for irregular, large, or cavity-containing foot wounds, as it promotes tissue formation and reduces microbial burden [71]. Nevertheless, NPWT is expensive and applicable only to specific wound locations. Copper-impregnated dressings, such as MedCu, on the other hand, have emerged as cheaper and more flexible alternatives to support wound healing. In this study, MedCu achieved a higher rate of complete wound closure than NPWT (48% vs 35%) with a comparable average time to closure [72]. Moreover, both patients and physicians preferred MedCu over NPWT (*p* < 0.001 and *p* < 0.005, respectively). Treatment costs were also significantly lower with MedCu ($470 vs $3360). Overall, this study supports the use of MedCu as a first-line therapy for diabetic foot wounds, particularly in cases where NPWT would otherwise be indicated, offering reduced medical costs, greater convenience, improved patient compliance, and comparable or superior clinical outcomes.

These conclusions have been further supported by a series of case studies involving hard-to-heal wounds of various origins and stages (Figs. 2c and 2 d), in which the MedCu dressing consistently enhanced wound healing and skin regeneration, resulting in rapid and uncomplicated recovery [73–75].

It is worth noting that, while clinical research on CuNPs (and derivatives) remains limited, a growing body of literature has explored the use of copper salts and copper-releasing materials in both preclinical and clinical settings. Those studies have identified that the therapeutic impact of copper treatments strongly depends on wound and exposure types [76]. For example, evidence from copper-releasing dressings and copper-based materials indicates that transient, locally controlled release of copper ions supports angiogenesis and granulation during impaired healing, such as in diabetic wounds [19, 74, 77]. Nevertheless, excessive or prolonged exposure, especially with rapidly dissolving formulations, can lead to oxidative stress and endothelial toxicity [55, 78, 79]. Hence, strong control over ion-release kinetics is desired.

### Copper nanoparticles as anti-inflammatory agents

As discussed in the previous sections, CuNPs can display anti-inflammatory and pro-angiogenic activities. Those partially arise from the crucial role of copper in biological processes regulating inflammation and the formation of new blood vessels. For example, copper is an integral part of certain antioxidant enzymes, such as superoxide dismutase, since they can neutralize reactive oxygen species [80, 81], which are drivers of inflammation and tissue damage. Copper colloids and ions have also been reported (in specific settings) to influence macrophage polarization, promoting the shift from pro-inflammatory M1 phenotype to the tissue-repairing M2 phenotype [82]. Moreover, copper can stabilize the hypoxia-inducible factor HIF-1α, promoting the overexpression of vascular growth factor [83, 84], a main driver of angiogenesis. Copper is also a cofactor of enzymes, such as lysyl oxidase, that support extracellular matrix stability [68, 85], enabling the growth of new blood vessels. Nevertheless, copper-mediated angiogenesis exhibits a biphasic dose–response: low-to-moderate levels promote endothelial proliferation, whereas excessive concentrations induce oxidative stress and cytotoxicity [86, 87]. Hence, the biological outcome of copper colloidal treatments is strongly governed by their ion release kinetics, requiring the need to maintain exposures within a defined therapeutic window.

Hand-foot syndrome, also known as palmar-plantar erythrodysesthesia, is a common dermatological toxicity associated with certain chemotherapeutic agents, such as capecitabine [88, 89], an oral prodrug of 5-fluorouracil that is widely used in the treatment of breast, gastric, and colorectal cancers [90]. Hand-foot syndrome results in erythema, swelling, and pain of the palms and soles, representing a dose-limiting side effect that often necessitates capecitabine dose reduction or interruption, potentially compromising therapeutic efficacy [91]. The condition seems to be (partially) caused by the extravasation of the cytotoxic agent from capillaries into the skin of pressure-bearing areas (feet and hands), leading to keratinocyte damage and inflammation responses.

A pilot clinical trial (NCT01291628) conducted at the Rabin Medical Center (Israel) explored the potential of copper oxide-impregnated fabrics, containing copper oxide microparticles produced by Cupron Inc. (Virginia, USA; US Environmental Protection Agency registration numbers 8454–2 and 8454–3), to prevent the hand-foot syndrome in cancer patients treated with capecitabine. The rationale was to harness the local anti-inflammatory and tissue-modulating properties of copper oxide particulates to mitigate chemotherapy-induced erythema, pain, and swelling. Previous attempts to prevent the condition with local agents such as pyridoxine or corticosteroids have yielded limited and inconsistent clinical benefit [92]. Although the copper-impregnated textile study was terminated in 2014, its results have not yet been published.

The same team from the National Health Service Trust (UK) that led the *ECHO* clinical trial also investigated the use of copper oxide particles for treating chronic venous diseases. The *Copper Impact on Venous Insufficiency and Lipodermatosclerosis (CIVIL)* study (NCT03283800) was a randomized, interventional clinical trial evaluating the therapeutic potential of a compression garment impregnated with copper oxide for patients with lipodermatosclerosis, a venous disorder characterized by inflammation and fibrosis of the subcutaneous fat, resulting in painful skin induration and plaques [93]. This condition often progresses into a chronic stage that precedes recurrent venous ulceration. Due to their high prevalence and limited treatment options, venous ulcers represent a major healthcare burden, accounting for an estimated loss of two million working days annually in the US and the UK combined [94].

After eight weeks of treatment, the copper oxide-based compression garment did not produce an overall reduction in swelling across the full cohort [95]. However, patients with acute disease did experience a significant decrease in leg swelling. Notably, the surface area of lipodermatosclerosis significantly improved with the copper treatment, decreasing from 42,637 mm^2^ to 35,739 mm^2^ (a 16% reduction). In contrast, the control group, which received a copper-free compression garment, showed worsening of the condition, with the affected surface area increasing from 41,487 mm^2^ to 43,210 mm^2^ (a 4% increase).

As previously discussed, copper-albumin colloids were the first commercial copper-based nanomedicines ever developed. Their clinical use was short-lived, limited mainly to the first quarter of the twentieth century, and they were ultimately abandoned in favor of more effective alternatives. Nevertheless, complexing copper with albumin offered several advantages, including stabilization of the metal by preventing precipitation and unwanted redox reactions, reduction of toxicity with safer delivery, and controlled release due to the slow, sustained dissociation of albumin-metal complexes. Moreover, albumin is a highly abundant human protein, offering excellent biocompatibility and reducing the immunogenic risk associated with treatment.

Given copper’s well-established anti-inflammatory, angiogenic, and collagen- and elastin-cross-linking activities, protein-stabilized colloidal copper formulations (e.g. copper ion-albumin complexes) have recently been revisited as topical treatments for alleviating the symptoms of osteoarthritis, a complex joint disease characterized by degenerative and inflammatory processes that lead to loss of articular cartilage and impaired mobility. A clinical trial (NCT03736109) exploring this approach was recently conducted at Assiut University (Egypt), and the results are still pending.

### Copper nanoparticles as photothermal agents for drug-resistant infections

Keratitis is an inflammatory disorder of the cornea that arises from irritation or infection [96]. It is often intensely painful, making the eye extremely sensitive to light and causing significant blurring of vision. If left untreated, this condition can progress quickly, leading to permanent corneal scarring and long-term impairment of vision [97]. Therefore, early intervention is essential to preserve visual function. This need is particularly urgent in infections caused by multidrug-resistant microorganisms, which tend to advance rapidly into severe cases.

Photothermal therapy is an emerging therapeutic modality that employs photothermal agents, often inorganic nanoparticles such as gold, to convert light into localized, high-intensity heat capable of ablating pathological tissue, such as tumors or infected regions, while sparing the surrounding healthy tissue [98, 99]. Unlike traditional drug-based treatments, photothermal therapy induces cytotoxicity effects through physical thermal stress rather than biochemical interactions. As a result, it has demonstrated promising efficacy against persistent or drug-resistant infections that fail to respond to conventional antibiotics [100].

Silver nanoparticles are well known for their broad antimicrobial activity [101, 102]. When doped with gold, the resulting nanostructures additionally display strong photothermal performance and enhanced absorption in the near-infrared region [29], a spectral window that enables deeper tissue penetration. Building on this principle, researchers from the Second Affiliated Hospital of Zhejiang University School of Medicine (China) developed a nanoconstruct composed of a hollow gold-silver core coated with a copper oxide shell (Figs. 2e and 2f), providing complementary photothermal and wound healing capabilities for the treatment of drug-resistant bacterial infections. In preclinical studies, these cupriferous hollow nanoshells successfully eradicated extended-spectrum *β*-lactamase *Escherichia coli* and methicillin-resistant *Staphylococcus aureus* infections (Figs. 2g and 2 h), while also promoting re-epithelialization in diabetic mouse wound models [103, 104]. Following these encouraging results, a phase I clinical trial was initiated in patients with drug-resistant keratitis (NCT05268718). In this study, the nanoshells were formulated into a gel (26.4 μg/mL) applied once to the ocular lesion, followed by 808-nm laser irradiation at 1.5 W/cm^2^, with temperature maintained at 40 °C for 10 min to avoid corneal damage. Although the trial was completed in 2024, its results have not yet been made public.

## Emerging therapeutic uses of copper nanoparticles at the preclinical level

In the last few years, there has been renewed interest in CuNPs, as their abilities to modulate biological mechanisms underlying diseases have been demonstrated. For instance, cuproptosis is a form of regulated cell death triggered by high intracellular copper levels that is mechanistically distinct from apoptosis, necrosis, and ferroptosis [105]. Unlike iron-overload ferroptosis, which is mediated by lipid peroxidation, cuproptosis is driven by the interaction between ionic copper and mitochondrial enzymes regulating the tricarboxylic acid cycle, resulting in loss of iron-sulfur cluster proteins and aberrant protein aggregations that collapse mitochondrial function and lead to cell death [106, 107]. Due to cancer cells’ greater sensitivity to copper accumulation, cuproptosis is emerging as a promising, relatively selective therapeutic strategy [108]. Accordingly, CuNP platforms, including solid copper cores, copper-based metal–organic frameworks, and self-assembled copper complexes, are being explored preclinically to increase copper levels in tumor cell populations and exploit their metabolic vulnerabilities, thereby overcoming resistance to conventional chemotherapeutics [109–111]. While promising, research on cuproptosis remains confined to preclinical studies. Substantial (pre)clinical validation, together with the resolution of key regulatory challenges (see Sect. "Lessons learned and translation framework for copper nanoparticles"), will be necessary before this new therapeutic modality can be regarded clinically feasible.

Beyond cuproptosis, copper is also fundamental in regulating cellular metabolism and immune system function [112–115]. As a result, it is an attractive target for metabolic and immunomodulatory interventions. For instance, copper is a key cofactor for enzymes modulating redox homeostasis, angiogenesis, and mitochondrial respiration. Thus, its dysregulation can be exploited to manipulate the metabolic profile of pathological tissues. Emerging therapeutic strategies include using CuNPs to increase stress in tumors or persistent infections [116, 117], while copper chelation is being explored to decrease copper levels and suppress proliferation and vascularization [118–120]. Furthermore, copper is being actively investigated to modulate immune cell activity [121]. However, those interactions are highly context-dependent, since CuNPs can polarize macrophages towards either the M1 (pro-inflammatory and anti-tumor) or M2 (anti-inflammatory and wound-healing) phenotypes, depending on the (pre)clinical setting, particle composition, surface chemistry, and physicochemical attributes [122, 123]. This immunomodulatory capacity suggests that CuNPs could be used to manipulate the tumor microenvironment toward pro-inflammatory and anti-tumor responses [82]. Nevertheless, the underlying mechanisms remain complex and difficult to precisely control, which currently limits their predictable therapeutic application.

Collectively, new approaches exploiting metabolic and immunological pathways are expanding the therapeutic potential of copper and CuNPs beyond traditional wound healing applications.

## Lessons learned and translation framework for copper nanoparticles

Although CuNPs can be easily synthesized at laboratory scale and are generally less expensive than many other inorganic nanomaterials, their translation to good manufacturing practice (GMP) production presents important challenges. The metallic copper cores are chemically dynamic and prone to oxidation and dissolution. As a result, small variations in oxygen exposure, processing conditions, or storage can alter the formulation’s physicochemical identity (e.g. Cu, Cu_2_O, or CuO) and its ion-release profile. In practice, this variability complicates batch-to-batch reproducibility, product equivalence, stability testing, and shelf-life definition. Copper microformulations may be easier to produce, but they typically exhibit broader size and shape distributions, further increasing heterogeneity and complicating quality control.

From a regulatory perspective, CuNPs are not an established class of nanomedicines. In contrast, iron oxide nanoparticles (e.g. ferumoxytol [44]) and certain inorganic nanoplatforms, such as hafnium oxide (e.g. Hensify [124]), have clinical precedents and defined evaluation pathways. As discussed above, most research involving CuNPs remains restrained at the preclinical or early translational stage, and the lack of class experience increases regulatory uncertainty. Advancement toward clinical translation will require a comprehensive understanding of copper speciation under physiological conditions (e.g. Cu^0^, Cu_2_O, CuO, ionic Cu^2+^), quantitative characterization of ion-release kinetics, and robust toxicological assessment, including immunoresponses, oxidative stress, and systemic effects. Particular attention will also be required for long-term retention and clearance, especially in highly perfused and fenestrated organs. While there are some approved copper-based drugs, such as copper histidinate, these are molecular formulations rather than nanoparticles. Nevertheless, they demonstrate that copper itself is not intrinsically disqualifying.

Following systemic administration, most CuNPs predominantly accumulate in the liver and spleen, consistent with sequestration by the reticuloendothelial system and hepatobiliary processing. This biodistribution profile highlights the need for detailed long-term pharmacokinetic and biopersistence studies. Importantly, nanoparticle dissolution and ion release make the systems more complex, as they can drive biological responses, such as the production of reactive oxygen species or protein binding, independently of the particulate biodistribution.

This contrasts with clinically established iron oxide nanoparticles, where macrophage uptake and gradual degradation ultimately contribute to a regulated endogenous iron pool, a fate that aligns with physiological metal homeostasis and supports certain approved indications (e.g. iron replacement therapy). For CuNPs, the long-term dissolution and their integration into homeostatic pathways are less clear. Unless copper-specific mechanisms, such as cuproptosis or targeted immunomodulation, are intentionally leveraged for therapeutic aims, persistent organ retention and uncontrolled ionic redistribution may represent another regulatory hurdle.

Overall, compared with clinically approved inorganic nanomedicines, CuNP development and translation require more precise control over chemical identity, dissolution behavior, and long-term fate. In the case of systemically administered nanoformulations, successful translation will likely depend on a clear clearance-by-design approach, where rapid degradation to safely excretable species and mechanisms of action justify the use of copper’s unique redox properties within a therapeutic aim.

## Conclusions

In recent years, interest in CuNPs and their therapeutic potential has been renewed. Although substantial research continues to develop new multifunctional copper-based nanoformulations across diverse preclinical applications, the clinical use of CuNPs remains constrained to a few well-defined areas, most notably wound healing and antimicrobial therapies, where the redox activity and biological properties of copper offer clear advantages. The formulations that have reached clinical practice are generally simple in composition, which facilitates manufacturing, scalability, and predictability of in vivo behavior, and they are typically applied topically to minimize systemic exposure and reduce risks associated with long-term accumulation. The recent discovery of cuproptosis has generated enthusiasm for the development of injectable copper formulations for targeted cancer treatment and immunotherapy; however, the extent to which these concepts will translate clinically remains uncertain and will require rigorous preclinical validation and deeper mechanistic understanding. Overall, despite more than a century of therapeutic use, copper nanomaterials have found their clinical niche primarily in localized applications, such as wound management, where robust evidence supports their efficacy. Other promising applications are still at an early stage of development, and their eventual clinical impact remains to be determined.

## Data Availability

No datasets were generated or analysed during the current study.
